# Ambivalent Food Craving and Psychobiological Characteristics in Individuals With Weight Suppression

**DOI:** 10.3389/fpsyg.2021.619025

**Published:** 2021-01-28

**Authors:** Mooah Lee, Jang-Han Lee

**Affiliations:** Department of Psychology, Chung-Ang University, Seoul, South Korea

**Keywords:** weight suppression, bulimic behavior, explicit-implicit, approach-avoidance, food craving, ambivalence

## Abstract

This study investigated the effects of psychobiological characteristics of non-obese women with a high level of weight suppression (H-WS) on explicit-implicit and approach-avoidance response toward food cues, depending on hunger-satiety states. The 634 participants were divided into two groups according to their weight history. If the difference between their highest weight over the last year and their current weight (a difference sustained at least for 1 year) was more than 5%, they were assigned to the “H-WS” group (*N* = 25). If the difference in weight was less than 5%, they were assigned to the “low level of weight suppression” (L-WS) group (*N* = 29). Explicit approach and avoidance toward food were measured by self-report questionnaires. Implicit approach and avoidance toward food cues were measured using an eye-tracker. Fasting blood samples were obtained to measure fasting serum leptin levels. After this, participants consumed a standard breakfast to control the satiety level. After breakfast, explicit-implicit approach-avoidance responses were repeatedly measured at the satiety states. Self-reported body shape concerns, drive for thinness, ambivalent food craving, and bulimic behavior were also assessed. The results showed that the H-WS group had lower leptin levels, and higher body shape concerns, drive for thinness, ambivalent food craving, and bulimic behaviors compared to the L-WS group. At the explicit level, the H-WS group reported lower approach and higher avoidance to food compared to the L-WS group, regardless of hunger-satiety state. Whereas, at the implicit level, the H-WS group showed higher approach during satiety rather than during hunger states. Regardless of the hunger-satiety state, there were no significant group differences with regard to implicit avoidance between the two groups. Thus, we confirmed that a high level of avoidance toward foods was observed in the H-WS group at the explicit level but not at the implicit level. Moreover, in contrast with a high level of explicit avoidance toward palatable foods, inhibition for implicit approach toward high-calorie foods seemed to be blunted after food consumption in the H-WS group. These inconsistencies may be associated with ambivalent food craving and vulnerability to bulimic behavior among H-WS individuals.

## Introduction

Weight suppression, which refers to weight loss and maintenance of reduced body weight, is widely acknowledged as a causal factor in the onset and maintenance of bulimic syndromes (Polivy and Herman, 1985; Fairburn et al., 1997; Stice, 2002; Keel and Heatherton, 2010). Bulimia nervosa is an eating disorder characterized by cyclic episodes of binge-eating episodes and compensatory behaviors (e.g., fasting, inappropriate/excessive exercise, and self-induced vomiting) to prevent weight gain (APA, 2013). A noticeable potential mechanism of the relationship between weight suppression and bulimic behavior is that individuals with weight suppression may be trapped in incompatible psychobiological states. Weight loss may lead to biological states prone to regaining weight, but also lead to psychological states urging a pursuit to thinness (Lowe et al., 2018). These biological and psychological states bind individuals with high level of weight suppression (H-WS) in bulimic behavior, which comprises cyclic binge-eating, and compensatory behaviors (Butryn et al., 2006; Herzog et al., 2010; Lowe et al., 2018) in the pursuit goals in opposite directions (i.e., promoting weight regain versus avoidance of weight regain). However, empirical evidence regarding this potential mechanism is, as yet, unavailable. Thus, the purpose of the present study was to investigate the effect of weight suppression on these contradictory biological and psychological states.

First, with regard to biological aspects, weight loss can lead to a heightened approach response toward high-calorie food, caused by decreased leptin levels (Bodell and Keel, 2015; Keel et al., 2017). A previous study showed that individuals with bulimia nervosa with a H-WS had higher self-reported weight gain concerns, but simultaneously, they showed a higher reinforcing value for chocolate candies (Bodell and Keel, 2015). Given that they showed increased behavioral activity to earn a large number of chocolates, it was concluded that they had increased approach tendencies toward such food, despite their conscious concern regarding weight gain.

Weight loss is accompanied by loss of adipose tissue, which is predominantly accountable for secreting leptin (Wolfe et al., 2004; Bodell and Keel, 2015). Thus, decreased leptin levels are often followed by weight loss (Sumithran et al., 2011). Leptin, a central appetite inhibition hormone (Schwartz et al., 2000; Morton et al., 2006), is involved in inhibiting responsivity of the reward circuit toward palatable food cues, and consequently contributes to reduction of food consumption (Coll et al., 2007). Further, leptin deficiency is associated with hyperphagia in rats (Sindelar et al., 1999) and severe early onset of adolescent obesity (Montague et al., 1997). Altered leptin levels after weight loss could promote weight regain (Rosenbaum et al., 2008; Sumithran et al., 2011) by modulating reward response to food cues (Morton et al., 2006; Farooqi et al., 2007; Grosshans et al., 2012). Therefore, we hypothesized that individuals with weight loss could have decreased leptin levels, and may thus have a heightened approach response toward high-calorie food cues.

Particularly, the effect of decreased leptin levels after weight loss seems to be influenced by the time point at which individuals lose weight. A 62-week follow-up study showed that decreased leptin levels after weight loss were sustained during approximately 12 months after weight loss (Sumithran et al., 2011). This means that the effect of weight loss on decreased leptin levels may be sustained for a certain period, rather than a permanent alternation. Thus, in this study, in order to examine the effect of weight loss on decreased leptin levels, a high level of weight loss group included individuals who experienced weight loss within the last 1 year.

However, with regard to psycho-behavioral aspects, individuals with weight loss may have a drive to maintain the state of reduced weight. A recent 20-year follow-up study showed that the vulnerability of individuals with weight suppression to developing bulimia nervosa is related to an elevated drive for thinness (Bodell et al., 2017). Moreover, individuals with weight loss showed elevated body shape concerns related to fear of weight regain (Lavender et al., 2015; Accurso et al., 2016). This indicates that, even if biological characteristics (e.g., decreased leptin levels) of individuals with weight suppression promote weight regain, these individuals may try to avoid regaining weight to maintain thinness. The drive for thinness seems to be aberrantly strong among individuals with a successful experience of weight loss. Lowe et al. (2018) describe this phenomenon as, “they revere thinness, they abhor weight gain.” Thus, a psychological response toward high-calorie food (e.g., avoidance of high-calorie food) may be totally contrary to biological characteristics (e.g., decreased leptin levels and approach toward high-calorie food). This inconsistency between biological characteristics and psychological response is important in order to understand how weight loss makes a person vulnerable to bulimic behavior.

According to a multidimensional model of craving, motivation toward appetitive cues can be represented by the two primary dimensions of approach and avoidance (McEvoy et al., 2004). That is, approach and avoidance responses can be distinguished as independent dimensions, or activated simultaneously; this phenomenon is called the “ambivalent states of craving” (Breiner et al., 1999). Addressing ambivalent food cravings in individuals with high weight suppression may elucidate the mechanisms that underpin the relationship between weight suppression and bulimic behaviors. Therefore, this study is possibly the first to apply the multidimensional model of craving to research on weight suppression.

Approach and avoidance toward food in individuals with high weight suppression may be different depending on the level of processing (i.e., explicit versus implicit level). According to the dual-process model of addictive behaviors (Wiers and Stacy, 2006), a reward response toward appetitive cues could emerge through two semi-independent systems, namely, the impulsive and reflective systems. The impulsive system (at the implicit level) comprises a quick, unintentional, and automatic reward response, whereas the reflective system (at the explicit level) involves a relatively deliberate, intentional, and consciously controlled reward response (Bargh, 1994). If the biological (i.e., approach) and psychological (i.e., avoidance) responses toward food have opposite directions in individuals with high weight suppression, it is possible that either of the two responses may be processed automatically rather than consciously.

Specifically, the effect of hormonal signals seems to be unconsciously processed within the impulsive system (van Honk et al., 2005; Bargh and Morsella, 2008). Hedonic neural activity toward food cues in the reward-related brain regions (i.e., non-homeostatic control of appetite) may be induced under the threshold of awareness (Takada et al., 2018), whereas avoidance of appetitive substances seems to be non-automatic and requires conscious effort (Tiffany, 1990). Therefore, we hypothesized that individuals with high weight suppression may show an automatic approach toward food cues at the urging of the impulsive system rather than the reflective system. Meanwhile, deliberate avoidance of food cues may emerge from the reflective rather than the impulsive system. The present study is the first to apply this dual-process model to research on weight suppression, for the purpose of examining vulnerability to bulimic behaviors.

To determine the effect of biological and psychological characteristics on food craving, the present study employed measurements for separate approach and avoidance responses toward food at the explicit and implicit levels. First, at the explicit level, a reflective response toward appetitive foods was measured using a self-report questionnaire. The Alcohol Approach Avoidance Questionnaire (AAAQ; McEvoy et al., 2004), which independently measures approach and avoidance responses in connection with the desire to consume alcohol, was modified to measure responses toward food consumption in the present study.

Second, at the implicit level, a modified attention task was used for measuring impulsive approach and avoidance toward food. We used an attention task with a pair of high- and low-calorie food cues that required specific behavioral responses, to identify the attentional approach and avoidance responses independently. In a previous study that used a pair of facial expressions (e.g., a paired sad and neutral face), the behavioral task consisted of an eye-tracking system to measure and distinguish the approach and avoidance attentional processes (Sanchez et al., 2013). In the present study, the implicit approach and avoidance attentional processes were measured by modifying the abovementioned behavioral task to one which utilized high- and low-calorie food cues.

Finally, approach-avoidance toward foods according to the level of hunger in individuals with high weight suppression is also important to observe. Inevitably, perception of and attention toward motivation-related cues are modulated by the physiological needs of the body (Goldstone et al., 2009). Thus, response toward food cues is regulated by the level of hunger, which is governed by the body’s desire to maintain homeostasis (Goldstone et al., 2009; Nijs et al., 2010). This phenomenon has been called hunger-satiety state-specific biased processing (Livneh et al., 2017). The strengthened approach toward food during hunger states and attenuated approach toward food during satiety is a natural reaction modulated by physiological needs.

However, individuals with high weight suppression may have blunted their inhibition to approach food even after they attain satiety, because the leptin signals communicating satiety may be insufficient in individuals with high weight suppression. Moreover, individuals with high weight suppression may experience difficulty in being consciously aware of their approach toward foods, because hormonal effects in the absence of hunger are likely to be unconsciously processed (McDuffie et al., 2004; Bargh and Morsella, 2008). If individuals with high weight suppression have blunted inhibition for implicit approach toward food even in the presence of perceived satiety, this may be a risk factor underpinning problematic binge eating. Therefore, this study is the first to examine blunted inhibition in the hunger-satiety state-specific biased processing in individuals with high weight suppression.

In summary, the present study sought to address the biological and psychological characteristics in individuals with high weight suppression (H-WS) to examine the relationship between weight suppression and bulimic behaviors, based on the ambivalence model of food craving. Specifically, the following hypotheses were tested. First, the H-WS group will report lower leptin levels and a higher level of drive for thinness, body shape concern, and bulimic behavior compared to the L-WS (low weight suppression) group. Second, at the explicit level, the H-WS group will report lower approach and higher avoidance responses toward food compared to the L-WS group, regardless of hunger-satiety state. Third, at the implicit level, the H-WS group will show a blunted inhibition of approach toward food cues during satiety compared to the L-WS group. Fourth, the H-WS group will report a higher level of ambivalent food craving compared to the L-WS group. Thus, the present study can prove to be helpful for a better understanding of the relationship between weight suppression and bulimic behaviors.

## Materials and Methods

### Participants

A total of 634 Korean women in their 20s voluntarily participated in an online Google survey about weight history, at the online university community and online diet community. Among the 634 participants, currently obese individuals [body mass index, BMI (weight in kg/height in m^2^) >30 kg/m^2^] (Kanazawa et al., 2002) were excluded from the study, since their appetite-related endocrine characteristics would differ from those of non-obese individuals (Myers et al., 2010). Additionally, individuals with the following latent factors that could influence altered appetite and weight control were excluded: at least one pregnancy experience, any amount of current smoking, diabetes, taking regular medication for medical conditions (e.g., thyroid disease), and consuming any appetite suppressant. Vegetarians were also excluded since the stimuli in the attention task included a picture of meat. Subsequently, based on their self-reported weight history, participants were assigned to one of two groups: H-WS group and low level of weight suppression (L-WS) group. The H-WS consisted of participants who reported intentionally losing weight and maintaining a reduced weight. Specifically, if the difference between their highest weight over the preceding year and their current weight (the difference sustained after weight loss) was more than 5%, they were assigned to the H-WS group. The L-WS group consisted of participants who reported maintaining a stable weight. Specifically, if the difference between their highest weight over the preceding year and their current weight was less than 5%, they were assigned to the L-WS group. Finally, 54 participants (H-WS group: *N* = 25; L-WS group: *N* = 29) were recruited to participate in our experiment.

### Measures

#### Leptin Levels

Serum leptin levels were measured after an overnight fast. Overnight fasting blood sampling was undertaken by a medical laboratory technologist. After collection of blood samples, the serum was transferred to cryotubes and stored in a freezer at −80°C. All serum parameters were analyzed by enzyme-linked immunosorbent assay using commercially available kits (Human Leptin ELISA kit, Millipore, Billerica, MA, United States) at the professional clinical laboratory services unit of a biotechnical company (Green Cross Reference Lab, Seoul, South Korea).

#### Ambivalent Food Craving Questionnaire

To assess subjective ambivalent food craving, the ambivalence subscale of the Stages of Change Readiness and Treatment Eagerness Scale (Miller and Tonigan, 1996) was modified, serving as a food version in the present study. The Ambivalent Food Craving Questionnaire (AFCQ) is a 19-item self-report questionnaire formulated to assess two-fold cognition for both food craving and problematic eating behaviors. The Cronbach’s alpha of the AFCQ in the present study was 0.75.

#### Approach and Avoidance of Food Questionnaire

The Approach and Avoidance of Alcohol Questionnaire (AAAQ; McEvoy et al., 2004) was modified to serve as a food version (Approach and Avoidance of Food Questionnaire; AAFQ) in the present study. The AAAQ is a 15-item self-report questionnaire designed to independently measure the strength of inclination “to drink” and “not drink” by employing a nine-point Likert scale ranging from “never” to “always.” The AAAQ has been modified to serve as a specific-food version (e.g., chocolate) in previous research (Cartwright et al., 2007; Cartwright and Stritzke, 2008). In the present study, the word “drink” was replaced by the word “eat” and the word “alcohol” was replaced by the word “palatable food.” For example, a sentence was modified from “I want to drink as soon as I have the chance” to “I want to eat as soon as I have a chance,” and from “I am avoiding places in which I might be tempted to drink alcohol” to “I am avoiding places in which I might be tempted to eat food.” Furthermore, the AAFQ provided both, the AAFQ-State (reporting the feeling at the present moment) as well as AAFQ-Trait (reporting one’s general characteristics) measurements. The Cronbach’s alpha of the AAFQ in the present study was 0.91.

#### Body Shape Questionnaire

The Body Shape Questionnaire (BSQ; Cooper et al., 1987) consists of 34 items assessing excessive concerns about body image and weight, using a 6-point Likert scale from “never” to “always.” The BSQ validated for use in Korean samples (Noh and Kim, 2005) was employed in the present study. The BSQ showed high internal consistency (Cronbach’s alpha = 0.96) in this study.

#### Eating Disorder Inventory-2

The drive for thinness subscale of the Eating Disorder Inventory-2 (EDI-2; Garner, 1991) was used in our study. This is a self-report questionnaire formulated to assess cognitive and behavioral characteristics in individuals with eating disorders, using a six-point Likert scale from “never” to “always.” The drive for thinness (DT) subscale (seven items) measures preoccupation with weight control and fear of weight gain. The internal consistency of the DT subscale in the present study was high (Cronbach’s alpha = 0.92).

#### Bulimia Test-Revised

The Bulimia Test-Revised (BULIT-R; Thelen et al., 1991) is a 28-item self-report questionnaire formulated to assess bulimic pathology; respondents are asked to choose one of five options describing the severity of their symptoms. The internal consistency of the BULIT-R in the present study was high (Cronbach’s alpha = 0.90).

#### Beck Depression Inventory

The Beck Depression Inventory (BDI; Beck et al., 1988) is a 21-item self-report questionnaire formulated to assess emotional, cognitive, and somatic symptoms of depression. Each item has four options that describe the degree of severity of the symptoms. The participant chooses the option which best describes their internal state during the past week. The internal consistency of the BDI in the present study was high (Cronbach’s alpha = 0.89).

#### State-Trait Anxiety Inventory

The State-Trait Anxiety Inventory (STAI; Spielberger et al., 1983) is a 20-item self-report questionnaire formulated to assess state (STAI-S) and trait anxiety (STAI-T) using a four-point Likert scale. The responses range from “not at all” to “so very much” for the STAI-S, and from “almost never” to “almost always” for the STAI-T. The internal consistency of the STAI in the present study was high (Cronbach’s alpha: STAI-S = 0.90; STAI-T = 0.90).

#### Perceived Hunger Level

To measure the participants’ perceived hunger level (PHL) before and after consuming breakfast, a 100 mm visual analog scale was used (0 mm: not at all hungry; 100 mm: as hungry as I have ever felt). Participants were asked to rate the following question: “How hungry do you feel at this moment?;” a higher score indicated a higher level of perceived hunger.

#### Attention Task

The implicit approach and avoidance attentional processes toward food cues were assessed by modifying the behavioral task used in a previous study, which utilized facial expressions to measure and distinguish approach and avoidance attentional processes (Sanchez et al., 2013). The present study modified used a modified version of this task by replacing facial expressions with high- and low-calorie food cues. An attention task was performed to directly record two different attentional components (approach and avoidance) toward high-calorie food cues. Images of food cues were selected from a food-pics image database (Blechert et al., 2014). First, 112 images were selected by the authors, based on their calorie level (high- and low-calorie foods) and familiarity of the food item in Korea. High- (e.g., pizza and hamburger) and low-calorie food cues (e.g., tomato and cucumber) were paired to match for color, complexity, brightness, and size as closely as possible (Blechert et al., 2014). Each pair was presented in a counterbalanced order, and cues were presented twice on the left and right side of the monitor.

**FIGURE 1:**
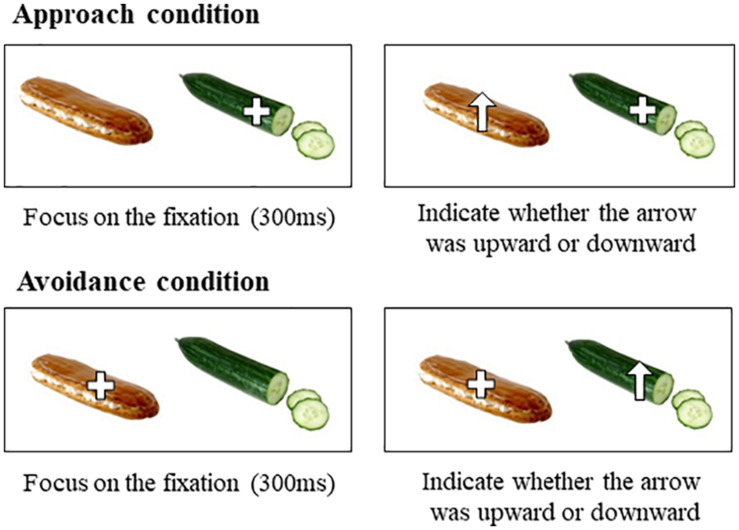
Paradigm of the attention task.

In the attention task (Figure 1), participants were instructed to focus on the fixation, and move their attention as quickly as possible toward that arrow and press one of two arrow keys on the keyboard to indicate whether the arrow was upward or downward. The attention task consisted of three different conditions. In the approach condition, fixation was provided on the low-calorie food cue for 300 ms and then, opposite foods cue appeared with an arrow (upward or downward arrow). Thus, the approach condition assessed how long participants took to shift attention from low-calorie food cues to high-calorie food cues. In the avoidance condition, fixation was provided on the high-calorie food cue for 300 ms, and then, opposite food cues appeared with an arrow (upward or downward arrow). Thus, the avoidance condition assessed how long participants took to disengage attention from high-calorie food cues and shift to low-calorie food cues. In the control condition, a fixation was provided on the left or right cue for 300 ms, and then, the fixation was replaced with an arrow (upward or downward arrow) in the same location as the fixation. The purpose of the control condition was to prevent participants from shifting their attention regardless of food cues by predicting the position of the arrow as being on the opposite side of the fixation. The attention task included a total of 100 trials that comprised 80 pairs of high- and low-calorie food cues and 20 pairs of neutral cues (e.g., furniture) as filler trials. Trials of approach and avoidance conditions for each cue (i.e., high-calorie and low-calorie food cues) were randomly presented. Both types of arrows appeared equally in the left and right positions in the approach and avoidance conditions. Trials of the control condition consisted of 10% of high- and low-calorie food cues and all neutral cues. Each cue of a pair was presented at a size of 80 mm × 100 mm, with their centers 200 mm apart, on a 23-inch monitor with a resolution of 1,920 × 1,080 pixels. During the attention task, participants’ eye movements were recorded by an eye-tracking system (Tobii TX300, Sweden; 300 Hz sampling rate). Data were processed using the Tobii studio (Tobii, Sweden). Areas of interest (AOIs) were designated for each trial and they corresponded with the size of each food cue (i.e., the locations of either high- or low-calorie food cues).

### Procedure

Participants were asked to avoid testing during their menstrual cycle because it could influence leptin levels (Ludwig et al., 2000; Ahrens et al., 2014). Participants were asked to abstain from eating food after waking up and before participating in the experiment. Participants arrived at the laboratory (Chung-Ang University, Seoul, South Korea) to measure their leptin levels at approximately 9:00 am following a 12-h overnight fast. When participants arrived at the laboratory, they filled out an informed consent form before participation, whereupon a blood sample was drawn to measure the leptin level. To confirm overnight fasting, blood glucose concentrations were also measured. All participants showed the expected level of fasting blood glucose concentrations. Each participant was then asked to sit and place their face approximately 60 cm from the computer monitor. To record eye movements, the eye-tracking system was calibrated. Subsequently, participants performed an approach-avoidance attention task. Then, participants were rated for AAFQ and perceived hunger. After that, participants ate a standard breakfast of a Gimbap (common and simple breakfast item in South Korea) of approximately 300 kcal (58 grams of carbohydrate, 7 grams of fat, 8 grams of protein, and 1 gram of sugar), to control their satiety level. After consuming breakfast, all procedures were repeated. Participants then completed questionnaires related to body, weight, and eating behavior. Finally, their body weight was measured in the laboratory. After completing the experimental procedure (which took approximately 1 h), participants received 35 dollars as a monetary reward and were fully debriefed regarding the experiment. The experimental procedures were approved by the Institutional Review Board of Chung-Ang University and the experiment was conducted according to the Declaration of Helsinki (Approval number: 1041078-201808-BRSB-165-01).

### Data Analysis

A minimum sample size per group (*N* = 20) for the present study was calculated using G^∗^Power 3.1 (Faul et al., 2009) for the repeated measures ANOVA, within-between interaction: 2 (condition: approach, avoidance) × 2 (group: H-WS and L-WS) using an alpha error probability of 0.05 (two-tailed), power of 0.80. Correlation among repeated measures (response scores in the approach and avoidance conditions in the attention task) was 0.07. A small to medium effect size (partial η*^2^* = 0.06) was expected, because a previous study reported medium to large effect sizes when they analyzed psychological differences between two groups with different levels of weight suppression (Bodell and Keel, 2015).

During the attention task, we analyzed eye-tracking data to verify that fixation actually occurred before a shift in attention toward an arrow, in the approach and avoidance conditions. For example, we verified whether fixation had occurred on the low-calorie food cues in the approach condition before presenting an arrow placed on the high-calorie food cues. Trials in which fixation did not occur were excluded from the analysis. On average, 24 trials were analyzed for each approach and avoidance condition. Next, the initial fixation latency for high-calorie food cues from fixation on low-calorie food cues in the approach condition was calculated. Further, initial fixation latency for low-calorie food cues from fixation on high-calorie food cues in the avoidance condition was also calculated. Since data for initial fixation latency in the attention task was positively skewed, response scores were calculated by means of a reciprocal transformation (De Muth, 2014). The higher the response score, the higher the response tendency. For example, a higher score in the approach condition indicated a higher level of approach toward high-calorie foods.

First, to examine group characteristics, an independent samples *t*-test was conducted to compare group differences in leptin levels, eating problems, and mood. Second, a 2 (group: H-WS and L-WS) × 2 (condition: approach and avoidance) × 2 (hunger level: hunger and satiety) repeated measures ANOVA was conducted on responses to the AAFQ in order to compare group differences between approach and avoidance at the explicit level. Moreover, a 2 (group: H-WS and L-WS) × 2 (condition: approach and avoidance) × 2 (hunger level: hunger and satiety) repeated measures ANOVA on the response score in the attention task was employed to compare group differences between approach and avoidance at the implicit level. Third, the response score of the approach and avoidance conditions in the attention task and the scores of the approach and avoidance subscale of the AAFQ were normalized with range 0 to 1 by min-max normalization (Jain et al., 2005; Jain and Bhandare, 2011) to compare explicit and implicit levels within identical range. In addition, a 2 (group: H-WS and L-WS) × 2 (condition: approach and avoidance) × 2 (hunger level: hunger and satiety) × 2 (processing level: explicit and implicit) repeated measures ANOVA was conducted. All analyses were performed using IBM SPSS version 25.0.

## Results

### Group Characteristics

As shown in Table 1, results from independent *t*-tests indicated that there were significant differences between the two groups in the amount of weight change for last year [*t*(52) = 6.96, *p* < 0.001, *Hedges*’ *g* = 1.97). There were no other significant group differences in age, height, current body weight, current BMI, and PHL. Also, there were significant group differences in level of depression [*t*(52) = 2.16, *p* = 0.037, *Hedges*’ *g* = 0.60] and state anxiety [*t*(52) = 2.15, *p* = 0.036, *Hedges*’ *g* = 0.59]. Consistent with the hypothesis, the H-WS group exhibited lower leptin level [*t*(52) = −3.63, *p* = 0.001, *Hedges*’ *g* = 0.99), higher body and shape concerns [*t*(52) = 5.78, *p* < 0.001, *Hedges*’ *g* = 1.56], drive for thinness [*t*(52) = 6.42, *p* < 0.001, *Hedges*’ *g* = 1.75], and higher level of bulimic behavior [*t*(52) = 5.07, *p* < 0.001, *Hedges*’ *g* = 1.38] than L-WS group. See Table 1 for further group details.

**TABLE 1:** Mean (SD) of demographics, body and eating-related characteristics of the high and low levels of weight suppression groups.

	**H-WS group (*N* = 25)**	**L-WS group (*N* = 29)**	***t* (52)**
Age	21.24 (2.07)	22.31 (2.36)	–1.76
WS level (kg)	7.46 (4.73)	0.95 (1.03)	6.96***
Highest weight (kg)	61.98 (8.14)	53.93 (3.46)	4.60***
Current weight (kg)	54.52 (5.15)	53.18 (3.57)	1.12
Height (cm)	161.92 (7.01)	161.63 (4.02)	0.19
BMI	20.25 (1.04)	20.16 (1.01)	0.31
Leptin (ng/ml)	13.90 (7.14)	22.47 (9.73)	−3.63**
PHL – Pre	28.3 (4.71)	27.97 (5.75)	0.23
PHL – Post	10.18 (6.07)	10.71 (19.48)	–0.13
BSQ	122.52 (26.19)	84.89 (22.05)	5.78***
EDI – DT	34.88 (5.88)	23.76 (6.73)	6.42***
BULIT-R	72.64 (22.29)	49.28 (10.17)	5.07***
BDI	10.64 (8.92)	6.14 (5.86)	2.16*
STAI – T	64.44 (11.23)	59.66 (10.78)	1.60
STAI – S	46.60 (12.55)	39.97 (10.13)	2.12*

*p < 0.05, **p < 0.01, ***p < 0.001.

*H-WS, High Weight Suppression; L-WS, Low Weight Suppression; WS level (kg), Difference Between 1-year Highest Ever Weight and Current weight; Highest Weight, (1-year Highest Weight; BMI, Body Mass Index; PHL, Perceived Hunger Level; BSQ, Body Shape Questionnaire; EDI – DT, Drive for Thinness Subscale of Eating Disorder Inventory; BULIT-R, Bulimia Test Revised; BDI, Beck Depression Inventory; STAI, State (S) Trait (T) Anxiety Inventory.*

### Approach, Avoidance, and Ambivalent Food Craving

To examine group differences in approach and avoidance trait toward food, independent *t*-test on each subscale score of AAFQ (T) were performed. There was no significant group difference on the trait approach subscale [*t*(52) = −0.65, *n.s.*], but there was significant group difference on the trait avoidance subscale [*t*(52) = 3.32, *p* = 0.002, *Hedges*’ *g* = 0.94]. The H-WS group reported significantly higher explicit avoidance compared to the L-WS group. To prove group difference on the ambivalent food craving, AFCQ was also analyzed. Consistent with hypothesis, group differences on AFCQ [*t*(52) = 3.28, *p* < 0.001, *Hedges*’ *g* = 0.89] were significant. That is, the H-WS group consistently showed a higher level of ambivalent food craving than the L-WS group at the different three questionnaires. See Table 2 for further group details.

**TABLE 2:** Mean (SD) of approach and avoidance toward food, ambivalent food craving of the high and low levels of weight suppression groups.

	**H-WS group (*N* = 25)**	**L-WS group (*N* = 29)**	***t* (52)**
AAFQ (T) – AP	6.54 (1.61)	6.78 (1.12)	−0.65
AAFQ (T) – AV	4.71 (2.18)	3.03 (1.14)	3.42**
AFCQ	10.84 (3.46)	8.07 (2.75)	3.28**

**p < 0.01.

*AAFQ (T), Approach (AP) and Avoidance (AV) of Food Questionnaire (Trait); AFCQ, Ambivalent Food Craving Questionnaire.*

### Psychobiological Characteristics and Ambivalent Food Craving

Consistent with the hypothesis, there were significant negative correlation between level of weight suppression and leptin (*r* = −0.45, *p* = 0.001), significant positive correlation between level of weight suppression and body/shape concerns (*r* = 0.55, *p* < 0.001), drive for thinness (*r* = 0.60, *p* < 0.001), and avoidance trait (*r* = 0.43, *p* = 0.001). Leptin level was negatively correlated with bulimic behaviors (*r* = −0.28, *p* = 0.041) drive for thinness (*r* = −0.34, *p* = 0.012). Bulimic behavior showed strong positive correlation with weight/body characteristics. Bulimic behavior is significantly correlated with body/shape concerns (*r* = 0.84, *p* < 0.001), with drive for thinness (*r* = 0.74, *p* < 0.001), weight suppression (*r* = 0.51, *p* < 0.001). Furthermore, bulimic behavior is significantly correlated with ambivalent food craving (*r* = 0.78, *p* < 0.001).

### Hunger-Satiety States Specific Explicit Approach and Avoidance Toward Food

**FIGURE 2:**
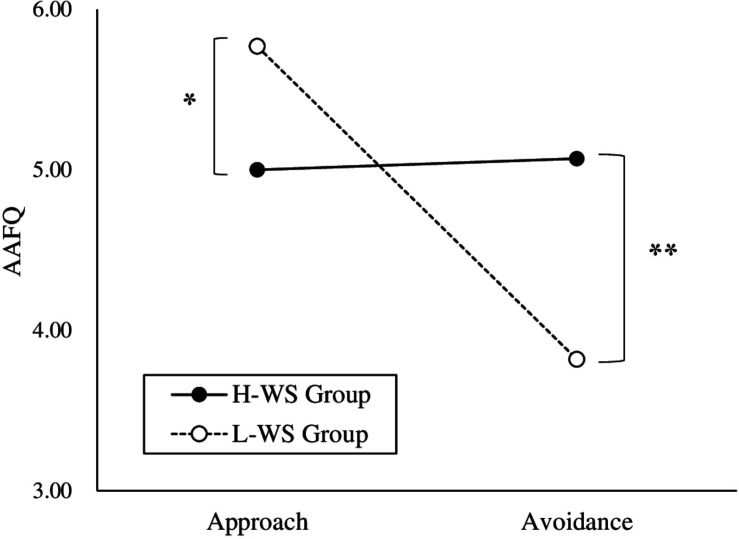
Explicit Approach and Avoidance of the High and Low Levels of Weight Suppression Groups (*p < 0.05, **p < 0.01). H-WS Group, High Weight Suppression Group; L-WS Group, Low Weight Suppression Group.

To examine whether explicit approach and avoidance differed by perceived hunger states, a 2 × 2 × 2 repeated measures ANOVA was conducted on the mean of the subscale in the AAFQ with group (H-WS and L-WS) as the between-subjects variable and subscale (approach and avoidance) and hunger level (hunger and satiety) as the within-subjects variable. There was no significant interaction between the H-WS and L-WS groups, subscale, and hunger states [*F*(1,52) = 0.15, *n.s.*]. There was a significant interaction between group and subscale [*F*(1,52) = 7.93, *p* = 0.007, η*_*p*_*^2^ = 0.13; Figure 2]. That is, regardless of hunger level, the H-WS group showed lower score at the approach subscale than the those of the L-WS group [*F*(1,52) = 4.40, *p* = 0.041, η*_*p*_*^2^ = 0.08] and higher score at the avoidance subscale than the those of the L-WS group [*F*(1,52) = 9.33, *p* = 0.004, η*_*p*_*^2^ = 0.15]. This means that the H-WS group reported higher explicit avoidance and lower explicit approach than the L-WS group, regardless of hunger-satiety states. There was a significant interaction between subscale and hunger level [*F*(1,52) = 60.13, *p* < 0.001, η*_*p*_*^2^ = 0.54]. That is, all participants showed the higher score at the approach than the avoidance subscale at the hunger states regardless of the group [*F*(1,53) = 47.23, *p* < 0.001, η*_*p*_*^2^ = 0.47]. The main effect of hunger level [*F*(1,52) = 4.70, *p* = 0.035, η*_*p*_*^2^ = 0.08] and subscale [*F*(1,52) = 6.84, *p* = 0.012, η*_*p*_*^2^ = 0.12] were also significant.

### Hunger-Satiety States Specific Implicit Approach and Avoidance Toward Food

**FIGURE 3:**
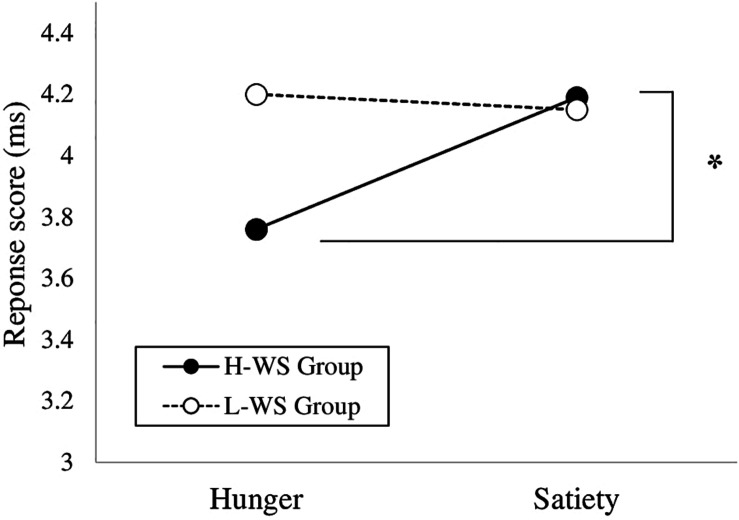
Approach Condition in the Attention Task of the High and Low Levels of Weight Suppression Groups According to Hunger Level (*p < 0.05).

To examine whether implicit approach and avoidance differed by perceived hunger states, a 2 × 2 × 2 repeated measures ANOVA was conducted on the implicit approach and avoidance scores of the attention task with group (H-WS and L-WS) as the between-subjects, and condition (approach and avoidance) as well as hunger level (hunger and satiety) as the within-subjects variable. As expected, there was a significant interaction effect between the H-WS and L-WS group, condition and hunger level [*F*(1,52) = 4.47, *p* = 0.039, η*_*p*_*^2^ = 0.08]. As a follow-up test up on the significant interaction of the three-way ANOVA, separate 2 (group) × 2 (hunger level) ANOVAs were conducted. For the approach condition, there was a significant interaction between group and hunger level [*F*(1,52) = 4.23, *p* = 0.045, η*_*p*_*^2^ = 0.08]. Within the H-WS group, the implicit approach score during satiety states was higher than those during hunger states [*F*(1,24) = 9.50, *p* = 0.005, η*_*p*_*^2^ = 0.28]. It means that the H-WS group showed increased implicit approach after consuming breakfast than 12-h fasting states (Figure 3). The main effect of group and the main effect of condition were not significant. There was no significant difference on the implicit approach score in the L-WS group according to hunger-satiety level. For the avoidance condition, there were no significant effects in the follow-up tests.

### Hunger-Satiety States Specific Explicit-Implicit and Approach-Avoidance Toward Food

Additionally, the difference approach-avoidance response between explicit and implicit level was investigated with an exploratory analysis. To examine whether explicit-implicit approach-avoidance differed by perceived hunger states, a 2 × 2 × 2 × 2 repeated measures ANOVA with group (H-WS and L-WS) as the between-subjects, and processing level (Explicit and Implicit), response direction (Approach and Avoidance) as well as hunger level (Hunger and Satiety) as the within-subjects variables was conducted on the normalized score of AAFQ and the attention task.

There was no significant interaction between the H-WS and L-WS groups, processing level, response direction, and hunger level [*F*(1,52) = 1.89, *n.s.*]. There was a significant interaction among group, processing level, and response direction [*F*(1,52) = 5.79, *p* = 0.02, η*_*p*_*^2^ = 0.10]. As a follow-up test up on the significant interaction of the three-way ANOVA, separate 2 (group) × 2 (processing level) ANOVAs were conducted. For the approach response, there was no significant interaction between group and processing level [*F*(1,52) = 0.52, *n.s.*]. For the avoidance response, there was a significant interaction between group and processing level [*F*(1,52) = 7.33, *p* = 0.009, η*_*p*_*^2^ = 0.12]. The H-WS group showed a higher level of avoidance than the L-WS group with the explicit process [*F*(1,52) = 9.35, *p* = 0.004, η*_*p*_*^2^ = 0.15], but there was no group difference with the implicit process. It means that group differences on avoidance response toward foods were observed not at the implicit level but only at the explicit level. While the H-WS group had higher explicit avoidance than the L-WS group, there was no group difference in implicit avoidance.

## Discussion

This study aimed to examine the biological and psychological characteristics related to weight suppression. Consistent with the hypotheses, the H-WS group showed lower leptin levels as well as a higher level of body shape concerns and a higher drive for thinness compared to the L-WS group. The H-WS group also reported a higher level of ambivalent food craving compared to the L-WS group. At the explicit level, the H-WS group showed a lower approach and higher avoidance toward palatable foods compared to the L-WS group, regardless of hunger-satiety states. At the implicit level, approach toward high-calorie food cues in the H-WS group was further strengthened after consuming breakfast, compared to the state of hunger. Implicit avoidance of food among the two groups was not different, regardless of hunger-satiety states.

In the present study, we found that a high level of avoidance toward foods in the H-WS group was observed only at the explicit level but not at the implicit level. Moreover, in contrast to a high level of explicit avoidance toward palatable foods, inhibition for implicit approach toward high-calorie foods was blunted after food consumption, in the H-WS group. These inconsistencies may be associated with ambivalent food craving and vulnerability to bulimic behaviors among H-WS individuals.

These results are in line with previous findings in the field of addiction which showed that alcohol-dependent patients reported ambivalent motivation toward alcohol. Although the results showing an approach bias in alcohol-dependent patients were not always consistent (Barkby et al., 2012; Eberl et al., 2013), heavy drinkers who wanted to quit drinking reported avoidance responses toward alcohol at the explicit level, as measured by a self-report questionnaire; however, when they were exposed to alcohol-related cues, they showed a spontaneous and non-volitional approach response toward alcohol-related cues at the implicit level, as measured by an implicit association test (Ostafin et al., 2008). Thus, if heavy drinkers show an approach response toward alcohol despite their motivation to quit alcohol, it means that this approach may be automatically provoked when they are exposed to alcohol-related cues and it may be difficult to inhibit using explicit higher-order cortical processes, given that the automatic approach response is provoked by their sensitized dopaminergic reward circuit (Volkow et al., 2011). In other words, like heavy drinkers who want to quit drinking, H-WS individuals may simultaneously experience conflicting motivations (i.e., approach-avoidance) toward high-calorie food at the different processing levels (i.e., explicit-implicit) after food consumption.

Considering biological and psycho-behavioral characteristics, the results of this study are in line with the explanation that individuals with high weight suppression may be trapped in a bio-behavioral bind (Herzog et al., 2010; Lowe et al., 2018). Weight loss may lead to a biological drive to regain weight, while simultaneously leading to a psychological drive to pursue thinness (Lowe et al., 2018). That is, individuals with weight suppression earnestly desire to sustain their reduced weight level, but may undergo biological compensatory changes that seek to restore weight after achieving significant weight loss (Lowe et al., 2011; Sumithran et al., 2011; Bodell and Keel, 2015). These biological and psychological characteristics lead individuals with a high level of weight loss toward bulimic behavior, that is, a cycle of binge eating and compensatory behaviors (Butryn et al., 2006; Herzog et al., 2010; Lowe et al., 2018), in pursuit of mutually contradictory goals. The present study is the first to provide empirical evidence of inconsistent biological and psychological characteristics in individuals with high weight suppression by virtue of having developed the ambivalence of food craving model.

Notably, the H-WS group displayed bluntly inhibited their implicit approach toward foods during satiety. This the altered hunger- or satiety-specific biased processing of the H-WS group may be related to their biological characteristics (i.e., altered leptin level). Similarly, leptin-deficient patients exhibited excessive neural responses to food cues even though they felt satiety and these excessive food reward responses decreased after a leptin replacement treatment protocol (Baicy et al., 2007). Future studies must include a focus on the relationship between hormonal alterations and altered cognitive and behavioral processing toward food cues to understand why H-WS individuals are vulnerable to bulimia nervosa.

One promising application of the results of this study is that it can assist in the formulation of educational interventions designed to help H-WS individuals understand their biological and psychological characteristics. Indeed, educational interventions addressing weight suppression have been reported to promote recognition of the effects of weight suppression on the body and of the relationship between weight suppression and bulimic behaviors, both of which have proven to be effective in improving treatment effects (Juarascio et al., 2017). In line with this, the results of the present study are expected to help H-WS individuals understand their biological approach response to food, which they had been hitherto unaware of.

This study has several limitations which should be noted. First, H-WS individuals in our study were not those with severe and pathological bulimic symptoms. Second, the characteristics of the implicit approach of the H-WS group were meaningful but without a large effect size. This may actually mean a small effect, but it may also be due to a limitation in the attention task itself, given that it only estimated the level of implicit approach-avoidance to foods based on eye movement toward food cues. Future studies need to develop a task that directly assess approach-avoidance at the implicit level rather than indirectly estimating it. Third, the number of trials which were actually analyzed in the attention task was too small since we only analyzed those trials where the participant’s fixation actually occurred. Future studies can improve on these limitations by developing automated attention tasks wherein an arrow is conditionally presented only when the participant’s fixation is actually captured at the AOI of the food cues.

In summary, one potential explanation for the vulnerability to bulimic behavior observed among H-WS individuals is the emergence of ambivalent food craving, which is due to the psychobiological inconsistences between explicit-implicit and approach-avoidance tendencies toward high-calorie foods.

## Data Availability Statement

The raw data supporting the conclusions of this article will be made available by the authors, without undue reservation.

## Ethics Statement

The studies involving human participants were reviewed and approved by Chung-Ang university IRB. The patients/participants provided their written informed consent to participate in this study.

## Author Contributions

ML: conceptualization, methodology, validation, formal analysis, investigation, data curation, writing, and visualization. JHL: conceptualization, methodology, validation, resources, supervision, project administration, and funding acquisition. Both authors contributed to the article and approved the submitted version.

## Conflict of Interest

The authors declare that the research was conducted in the absence of any commercial or financial relationships that could be construed as a potential conflict of interest.
